# Foreign Summary

**Published:** 1838-10-10

**Authors:** 


### FOREIGN SUMMARY.

*Notice of the Hôtel Dieu of Paris.*—The Hôtel Dieu of Paris is probably the oldest hospital in Europe. It was founded about the year 660, for the refuge and assistance of pilgrims, and the sick and destitute of the metropolis. Its motto was, *medicus et hospes.* At certain seasons it was so crowded that we are told that, at the beginning of last century, there was sometimes upwards of 9,000 inmates, of all ages, sexes, and conditions in it, at one time. There were wards for lying-in women, children, venereal patients, and the insane. But, after the revolution, a great improvement in all these arrangements took place. The lunatics were transferred to the Salpetriere and Charenton Hospitals, and subsequently also to the Bicêtre; the Maison d'Accouchemens and the Maternité received the obstetrical cases, while the children were established in a separate hospital, Hôpital des Enfans Malades; a venereal hospital was set apart for syphilitic cases; the other hospitals of the metropolis, viz. Necker, Cochin, Beaujon, La Charité, La Pitié, St. Antoine, St. Louis, &c. were greatly enlarged and improved; and, lastly, a Central Board (Bureau Central) was instituted to superintend the distribution of the various patients.

By these arrangements the crowded state of the Hôtel Dieu was reduced to 1,800, 1,200, and lastly, to 1,000—which last number may be stated to be the average amount of patients in its wards at the present time.

The administrative service of this great hospital consists of a director, a treasurer, and four secretaries.

The medical staff, or "service de santé," consists of ten physicians, three surgeons, one chief clerk, *chef de clinique*, nine physicians' in-door clerks, *eleves internes en medecine*, ten surgeons' in-door clerks, 124 pupils, one apothecary and an assistant, and ten in-door pupils of pharmacy.

The sisters of charity, who act as nurses here, belong to the order of St. Augustin: of these, there are 32 matrons (*mères*,) and 12 novices.

The physicians visit their patients every morning at six o'clock, assisted by their "*internes*," and their "*externes*;" and the resident physician visits all the wards each evening. All cases of urgency and danger are attended to by two of the *internes*, who keep watch night and day.

Of all the hospitals in Paris, the Hôtel Dieu is that which is best supplied with water. The author of the paper, from which we draw our remarks, candidly admits however that Paris, when compared with London as to the supply of this necessary of life, is still "*dans l'enfance de l'art.*" It is generally supposed that London consumes upwards of 100 times as much water as Paris. The chief supply to the Hôtel Dieu is from the pump of Notre Dame. But it is not possible for us to follow the author through all his minute details of the general economy of this vast establishment. We must therefore refer our readers for further particulars to the number for last November of the *Annales d'Hygiene.*—*Med. Chirurg. Review.*

---

*On the Treatment of Fractures, by M. Velpeau.*—The object of the indefatigable surgeon of La Charité is to shew that a patient, after the broken limb has been secured in an "apparatus inamovible," may be permitted to walk about on crutches, with perfect safety.

He premises his remarks by observing that a very popular error still exists, that it is of great consequence that a fractured limb should be *set*, as soon after the occurrence of the accident as possible; and hence it is generally supposed, by unprofessional persons, that the sooner that the displaced ends of the bone are rectified the more favourable will be the progress of the case. "Unless," says M. Velpeau, "some serious complication exists, it is almost quite indifferent whether the apparatus be applied at the end of a few minutes or of the first twenty-four hours. Up to this time there is nothing to be dreaded from delay. I wish to insist much on this point, in order to prevent the unnecessary alarm in a family, which is excited on all occasions of a broken bone."

Some surgeons, however, carry this system of delay much too far, when they recommend that a fractured limb be left without any apparatus for the first eight or ten days. This period, they pretend to say, is necessary to combat the swelling and inflammatory action of the surrounding soft parts; and as the agglutinative and consolidative process of incipient ossification does not commence till the second week or so, it is useless, they add, to keep the ends of the bone in exact apposition.

M. Velpeau very properly condemns this line of practice. How can the sharp ends of the bone, he asks, remain in contact with the soft parts without irritating them, and exciting spasm and inflammation? and as for the objection that the compression from the immediate application of splints and bandages is apt to induce gangrenous inflammation, it holds good only of the injudicious, and not of the scientific, employment of the means.

Indeed, ever since the commencement of my practice, I have uniformly acted on the principle of immediately reducing the displaced bones, and of then surrounding the limb with discutient compresses, and with a moderately-compressive bandage, carefully applied from the fingers or toes upwards along the whole extent of the injured limb. These objects being effected, the only other indication was, in my opinion, to maintain the limb in an immoveable, and as easy a position as possible. By following these principles of practice, I (M. Velpeau) have succeeded in curing some most severe and unfavourable cases of fractured limbs, even when the bone had projected through the integuments, and great infiltration had taken place into the soft parts.

M. Velpeau gave a very fair trial to the practice recommended by Baron Larrey—of wetting the bandages, when first applied, with an agglutinative fluid composed of liq. plumbi, camphorated spirit, and the white of eggs—and was inclined for sometime to think very favourably of it. But of late years he has discontinued it, in consequence of the following objections:—

1. When the tumefaction of the limb subsides, a hollow is left between it and the bandage.

2. It is difficult to remove the bandage, in case such a removal be expedient.

The latter of these objections is, in a great measure, obviated by the proposal of M. Seutin, a distinguished surgeon of Brussels, to employ a mere solution of isinglass or of starch in place of the liquid of Barron Larrey; the former being as firmly agglutinative, and much more readily softened and removeable than the latter,

M. Velpeau approves of the suggestion; and the following is the mode in which he has availed himself of it in the apparatus which he now generally uses in the treatment of fractures.

Having reduced the displaced bone by appropriate extension, he applies a roller from the toes up to the knee: this is to be well wetted with a strong solution of starch; and then it is to be applied backwards from the knee down to the toes. The opposite surfaces of the roller become very quickly glued to each other, so that it is not at all necessary to use pins to keep any of the turns together.

It is a good plan to place some wadding along each side of the tendo-achillis, so as to fill up the hollow between the two layers of the bandage. He then applies two or three pasteboard splints, moistened in the starch solution, on each side of the limb, and one to the sole of the foot. These are maintained in strict apposition to the limb by means of another roller, which is also wetted with the liquid, so as to cause it to adhere firmly to the splint. When this has dried, in the course of two or three days, the limb is found to be cased in a solid greave, so to speak, of pasteboard and bandage, and all risk of the displacement of the fractured ends of the bone is effectually prevented. Hence the patient may not only move or turn the limb in bed with perfect safety, but he may even rise and walk about on crutches, whenever the desiccation of the apparatus is complete—and this is usually the case by the third or fourth day.

M. Velpeau assures us that he has treated upwards of sixty cases of fracture, in the manner now described, with most complete success, and with far less trouble, both to surgeon and patient, than with the ordinary apparatus. He alludes also to the great success of M. Seutin in Brussels, who has now for some years trusted entirely, in his extensive surgical practice, to the "methode par compression inamovible," in all cases of fracture of the upper and lower extremities.—*Ib. From L'Experience, Journ. de Med. et de Chirurg.*

---

*Proto-ioduret of Iron in Syphilis.*—This chalybeate has been of late employed with very decided advantage, internally as well as externally, in the treatment of old syphilitic cases, more especially in persons of a scrofulous or lymphatic constitution. It may be usefully combined with bitters, anti-scorbutics, &c., in the dose of from six to forty grains daily.

As an external remedy it may be employed as a lotion or injection.

The proto-ioduret of iron is obtained by heating together about fifty parts of iodine, and fifteen parts of iron filings, with a hundred parts of distilled water: the liquor acquires a greenish colour, It is to be filtered, and quickly evaporated to dryness, protecting it all the while as much as possible from contact with the atmospheric air.

The salt is to be kept in a firmly corked phial.—*Ib. From Revue Medicale.*

---

*Medical Portrait Gallery.*—This is a monthly publication, edited by Mr. Pettigrew, and devoted to memoirs of the most celebrated physicians, surgeons, &c. &c., who have contributed to the advancement of medical science. The numbers published, have respectively contained portraits of Æsculapius, Albinus, and Sir Henry Halford; Ruysch, Haller, and Sir Anthony Carlisle; Linacre, Akenside, and Sir Charles Clarke. The memoirs, generally, are highly spoken of, but the portraits of living characters are represented as drawn in too glowing colours.

